# The mitochondrial transport protein SLC25A43 affects drug efficacy and drug-induced cell cycle arrest in breast cancer cell lines

**DOI:** 10.3892/or.2013.2247

**Published:** 2013-01-23

**Authors:** MARIKE GABRIELSON, ELISABET TINA

**Affiliations:** 1 School of Health and Medical Sciences, Örebro University Hospital, SE-70185 Örebro, Sweden; 2 Clinical Research Centre, Örebro University Hospital, SE-70185 Örebro, Sweden

**Keywords:** SLC25A43, cytotoxicity, viability, cell cycle arrest, human epidermal growth factor receptor 2-positive breast cancer

## Abstract

The mitochondria have been identified as key players of apoptosis, cell proliferation and cell cycle regulation. However, the role of mitochondria in breast cancer and treatment failure remains unclear. We have previously shown a common deletion of the gene *SLC25A43* in human epidermal growth factor receptor 2 (HER2)-positive breast cancer. This gene is coding for a mitochondrial inner membrane transporter and, to date, little is known about the function of this protein. We have also found that low protein expression of SLC25A43 significantly correlates with a lower S phase fraction in HER2-positive breast cancer. The aim of this study was to investigate whether knockdown (KD) of *SLC25A43* could have an effect on the cytotoxicity of different cytostatic drugs using MCF10A, MCF7 and BT-474 cells. Following siRNA-mediated KD of *SLC25A43*, one non-malignant and two breast cancer cell lines were exposed to the anthracycline epirubicin or the taxane paclitaxel. The HER2-positive breast cancer cells were also exposed to the targeted therapy trastuzumab and dual exposure to trastuzumab and paclitaxel. We found that KD of *SLC25A43* resulted in a decreased cytotoxic effect of paclitaxel in the two cancer cell lines (P<0.05). Further analysis of cell cycle phase distribution showed that KD increased the paclitaxel-induced G2/M block in these two cell lines (P<0.05). KD of *SLC25A43* also reduced the inhibitory effect of trastuzumab on cell proliferation in the HER2-positive cancer cell line BT-474 (P<0.05), and the drug-induced G0/G1 block (P<0.05). Moreover, *SLC25A43* influenced the percentage of Ki-67-positive cells. Our findings demonstrate that the mitochondrial protein SLC25A43 affects drug efficacy and cell cycle regulation following drug exposure in breast cancer cell lines.

## Introduction

Although a number of cytostatic drugs with different mechanisms of action are used in the treatment of breast cancer, intrinsic or acquired resistance is a common problem culminating in the treatment failure (1). Taxanes and anthracyclines are widely used and their antitumour activity contributes to apoptosis in cancer cells through interaction via several intracellular functions (2,3). For instance, the taxane paclitaxel exerts its apoptotic effects by stabilising the microtubuli thus preventing them from disassembling (4). It is also a potent inhibitor of chromosomal replication resulting in late Gap 2 (G2) or mitotic (M) block of the cell cycle (5). Paclitaxel is generally used as a single agent as well as in combination therapy with other chemotherapeutic agents to treat early and advanced stage breast cancer (6). The reported overall response rate of paclitaxel is 21 to 54% when used as a first-line single agent therapy. Anthracyclines such as epirubicin, on the other hand, act in part by inhibiting DNA and RNA synthesis as well as by inducing permanent double strand DNA breaks by inhibiting the enzyme topoisomerase II thereby promoting G2-blockage (7,8). Anthracycline-based polychemotherapy reportedly reduces the mortality rate by 20 to 38% (9). However, anthracyclines are associated with a higher rate of acute and late adverse effects, such as increased risk of cardiotoxicity and secondary leukaemia (10).

In order to achieve an individualised antitumour therapy, targeted therapy directed against specific cellular molecules has been developed. The monoclonal antibody trastuzumab is the first targeted therapy directed against the extracellular domain of the human epidermal growth factor receptor 2 (HER2). Trastuzumab is often used in treatment of patients with an amplification/overexpression of HER2. Binding of trastuzumab to the HER2-receptor induces a G1 block, thus reducing both the proliferation and survival advantages of the tumours (11–15). Trastuzumab has a modest overall response rate ranging from 15 to 30% when used as a neoadjuvant single agent (16). In clinical settings, trastuzumab is predominantly administered in combination with chemotherapy drugs such as taxanes to improve disease-free and overall survival (17–19). Nevertheless, even when combining targeted therapy and chemotherapy, treatment failure remains an ongoing problem in the clinic, indicating the complexity of the mechanisms involved in drug efficiency.

In recent years, a hypothesis has emerged, identifying a new role of the mitochondria as an important regulator of the cell cycle by connecting mitochondrial function and energetic status with cell cycle progression (20–26). This supports the theory that altered mitochondrial function along with deregulated energy metabolism are key for cancer cells to sustain uncontrolled cell proliferation and avoid apoptosis (27).

We previously described novel findings of a commonly deleted gene in HER2-positive breast cancer (28). This gene encodes the mitochondrial inner membrane solute carrier protein SLC25A43. However, the complete function of SLC25A43 along with the substrate carried by this membrane transporter remains unknown. We noted that lower expression of SLC25A43 in the tumours correlated significantly with a lower S phase fraction in HER2-positive breast cancer (28).

The aim of this study was to investigate the possible role of the mitochondrial protein SLC25A43 in drug sensitivity *in vitro*. Our results showed that knockdown (KD) of *SLC25A43* in different breast cell lines altered the sensitivity to the cytostatic drugs, as demonstrated by altered cell viability and altered distribution and regulation of cell cycle phases. The findings presented herein support the theory of a mitochondrial role in drug susceptibility.

## Materials and methods

### Cell culturing

The immortalized breast epithelial cell line MCF10A, the HER2-negative breast adenocarcinoma cell line MCF7 and the HER2-positive breast cancer cell line BT-474 were all obtained from the American Type Culture Collection (Manassas, VA, USA). MCF10A was cultured in D-MEM/F-12 supplemented with 10% FBS, 10 μg/ml insulin, 20 ng/ml H-EGF and 0.5 μg/ml hydrocortisone. MCF7 was cultured in Eagle's minimum essential medium (MEM) supplemented with 10% FBS and 10 μg/ml insulin, and BT-474 was cultured in RPMI-1640 supplemented with 10% FBS and 10 μg/ml insulin. Cells were cultured in a humidified atmosphere at 37°C with 5% CO_2_.

The cells were seeded at a density of 25×10^3^ cells/cm^2^, 24 h before transfection. Transfection was performed using Lipofectamine™ 2000 (Invitrogen, Carlsbad, CA, USA) and scrambled siRNA (siCtrl) or target-specific siRNA [Hs_LOC203427_2 (Qiagen Sciences, Germantown, MD, USA)] (siSLC) for KD, according to the manufacturer's recommendations. The obtained *SLC25A43* mRNA KD was 90% in MCF10A, 90% in MCF7 and 75% in BT-474, and was stable in all cell lines for a minimum of 96 h.

For all cytotoxicity assays, cells were exposed to the drugs 24 h after transfection and incubated for 72 h, using 16 or 160 nM paclitaxel or 2.5 or 10 μM epirubicin (Actavis, Hafnarfjordur, Iceland). BT-474 cells were also subjected to exposure of 10 or 100 μg/ml trastuzumab (Roche AB, Stockholm, Sweden) or a combination of trastuzumab (10 μg/ml) and paclitaxel (16 nM), referred to as T/P. As a drug-free control for all experiments, cells were transfected and cultured in medium without cytostatic drugs. Incubation with epirubicin was terminated after 1 h by replacing the medium with fresh medium.

### Flow cytometry assays

#### Determination of viable cells

Cell viability was determined by incubating collected cells in the culture medium together with the trypsinized cells using 0.25 μg 7-AAD (BD Biosciences, San Jose, CA, USA) for 10 min at room temperature and protected from light, according to the manufacturer's protocol.

#### Inhibition of cell proliferation assay

Measurement of cell proliferation was carried out using PKH67 Green Fluorescent Cell Linker (Sigma-Aldrich, St. Louis, MO, USA) according to the manufacturer's protocol. PKH67 is a green fluorochrome that incorporates into the cell membrane without affecting cell viability. Following cell division, fluorescence intensity is decreased due to dilution of the fluorochrome. The cells were stained with PKH67 at time of seeding.

#### Cell cycle phase analysis

Analysis of cell cycle phase distribution was performed as previously described (29) on isolated cell nuclei using 100 μg/ml propidium iodide (PI) (Sigma-Aldrich) for DNA-staining.

#### Cell cycle regulation assay with Ki-67 and p21

The expression of Ki-67 and p21 was analysed after 72 h of exposure with 16 nM paclitaxel, 2.5 μM epirubicin, 10 μg/ml trastuzumab or a combination of trastuzumab (10 μg/ml) and paclitaxel (16 nM), as indicated. Pelleted cells were resuspended for 10 min with an ice cold lysing solution containing 0.1% Igepal CA-630 in wash buffer (1% FBS in PBS) to isolate cell nuclei. The nuclei were then washed once with ice cold Wash buffer before adding antibodies against p21 Alexa Fluor^®^ 488 (1:50, clone 12D1; Cell Signaling Technology, Inc., Danvers, MA, USA) and Ki-67 PE (clone 56; BD Biosciences) to one tube and isotype control to a second tube [IgG isotype for Alexa Fluor 488 (1:50) and IgG1 isotype for PE]. All tubes were supplemented with 0.5 μg 7-AAD (BD Biosciences) for DNA staining and incubated for 15 min. The nuclei were then diluted with PBS and stored on ice prior to analysis. All incubation steps were performed on ice.

The flow cytometry analyses were performed 96 h after transfection using an EPICS Altra equipped with an argon laser (488 nm) and EXPO 32 software (Beckman-Coulter, Fullerton, CA, USA). During acquisition, 10,000 events were collected. For analysis of cell viability, PKH67 and the expression of cell cycle proteins, Kaluza software (Beckman-Coulter) was used. Cell cycle phase distribution was analysed using ModFit LT v3.2 (Verity Software House, USA). To analyse the expression of Ki-67 and p21 a gate was set on the 7-AAD histogram to exclude sub-G0/G1 events and then the isotype control was used to determine the cut-off for positivity. The positivity was expressed as a percentage of positive cells.

#### Statistical analysis

The Mann-Whitney test was used to assess statistical significance in all assays. P<0.05 was considered to indicate a statistically significant difference. SPSS 17.0 statistical software for Windows (SPSS Inc., Chicago, IL, USA) was used for all tests.

## Results

### Reduced paclitaxel efficacy following SLC25A43 knockdown

**Figure 1:**
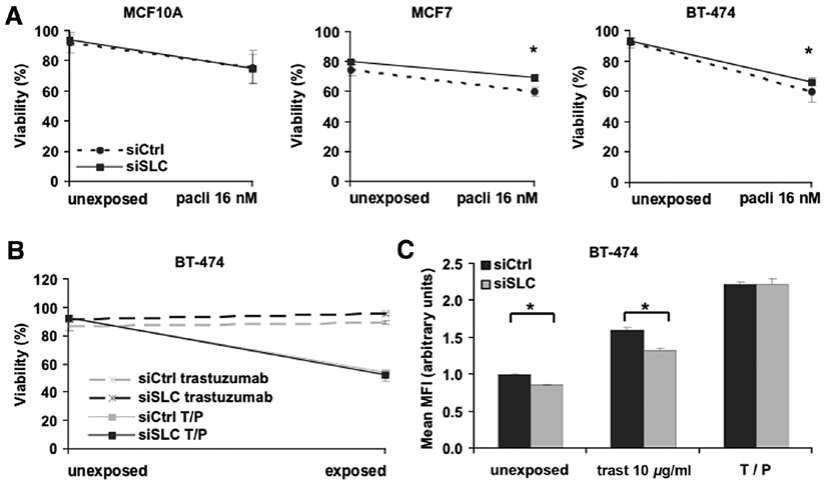
*SLC25A43* KD reduces the cytotoxic effect of paclitaxel and trastuzumab in breast cancer cells. (A) KD of *SLC25A43* 24 h before exposing cells to 16 nM paclitaxel for 72 h significantly increased viability in the MCF7 and BT-474 cancer cells but not in the non-malignant mammary epithelial cells MCF10A, compared to control siRNA. (B) Viability of BT-474 with or without *SLC25A43* KD after 72 h exposure with 16 nM paclitaxel, 10 μg/ml trastuzumab or combination of paclitaxel and trastuzumab (T/P) (16 nM and 10 μg/ml). Viability after T/P exposure was independent of *SLC25A43* KD. (C) Trastuzumab-induced inhibition of cell proliferation, measured using PKH67, was significantly reduced after *SLC25A43* KD. However, when combining trastuzumab and paclitaxel (16 nM and 10 μg/ml), the drug-induced inhibition was independent of *SLC25A43* KD. For all assays, three experiments were performed in duplicates. The data are expressed as the means ± SD. ^*^P<0.05.

Our experiments described the effects of SLC25A43 on drug-related cellular outcome and sensitivity utilising siRNA-mediated mRNA KD in *in vitro* models. The non-malignant cell line MCF10A and the two cell lines MCF7 and BT-474, with different clinically relevant breast cancer phenotypes, were used. To evaluate the effects of *SLC25A43* KD on cytotoxicity, we analysed the viability of MCF10A, MCF7 and BT-474 cells after 72-h exposure to paclitaxel or epirubicin. The sensitivity for paclitaxel was not altered in MCF10A cells after KD of *SLC25A43* (Fig. 1A). By contrast, KD of *SLC25A43* resulted in a significant reduction of cytotoxic effects by 16 nM paclitaxel in the two breast cancer cell lines MCF7 and BT-474 compared to siCtrl (Fig. 1A). The reduction in cytotoxicity was also observed at the higher concentration of paclitaxel in MCF7 cells (P<0.05). Contrary to the paclitaxel exposure, KD of *SLC25A43* did not influence the cytotoxic effect of epirubicin in any cell line (data not shown).

The HER2-positive breast cancer cell line, BT-474, was further investigated through exposure with the targeting drug trastuzumab. Trastuzumab alone does not directly induce cell death *in vitro;* however, it inhibits proliferation and is an important agent when treating HER2-positive breast tumours (30). We therefore measured both the viability and inhibition of proliferation of BT-474 cells subjected to exposure of either trastuzumab (10 μg/ml) or a combination-dose of trastuzumab (10 μg/ml) and paclitaxel (16 nM) (T/P) (Fig. 1B and C). As expected, trastuzumab alone did not induce cell death (Fig. 1D). However, the reduced cytotoxic effect of paclitaxel after *SLC25A43* KD was no longer present when paclitaxel was combined with trastuzumab. Measuring proliferation of BT-474 cells after trastuzumab exposure revealed that *SLC25A43* KD contributes to a significantly lower inhibition of cell proliferation (Fig. 1C). This effect was, however, eliminated when combining trastuzumab and paclitaxel. These data show that KD of *SLC25A43* influences the efficacy of paclitaxel and trastuzumab in the breast cancer cell lines.

### SLC25A43 affects the cell cycle phase distribution upon drug exposure

Paclitaxel, epirubicin and trastuzumab are all known to induce cell cycle arrest, thus leading to cell death or inhibition of proliferation. To evaluate a possible influence of *SLC25A43* KD on the cell cycle, we analysed the cell cycle phase distribution in surviving cells following drug exposure.

**Figure 2:**
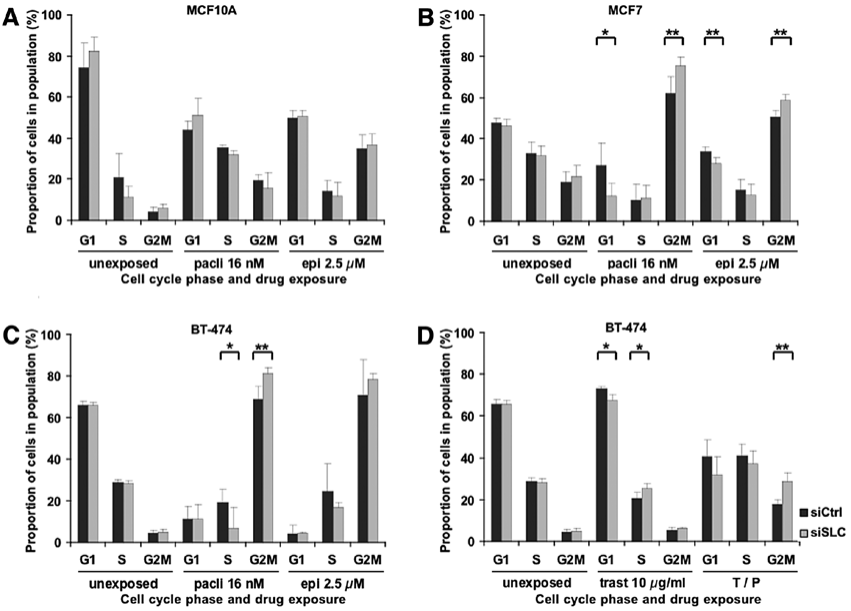
*SLC25A43* KD significantly alters the cell cycle phase distribution after drug exposure in the breast cancer cell lines. (A-C) Cell cycle phase distribution in (A) MCF10A, (B) MCF7 and (C) BT-474 cells after 16 nM paclitaxel or 2.5 μM epirubicin exposure for 72 h, comparing *SLC25A43* KD with control siRNA. Both paclitaxel and epirubicin induced G2M block in all three cell lines, and KD of *SLC25A43* significantly increased the paclitaxel-induced block in MCF7 and BT-474 cells. Only in MCF7 did *SLC25A43* KD significantly alter the epirubicin-induced G2M block. (D) Cell cycle phase distribution in BT-474 after 72 h of 10 μg/ml trastuzumab or trastuzumab-paclitaxel double exposure (T/P) (16 nM and 10 μg/ml), comparing *SLC25A43* KD with control siRNA. Exposure with trastuzumab induced a G1 block that was significantly reduced in *SLC25A43* KD cells. *SLC25A43* KD prior to double exposure significantly increased the paclitaxel-induced G2/M block. For all assays, three experiments were performed in duplicates. The data are expressed as the means ± SD. ^*^P<0.05 and ^**^P<0.01.

Paclitaxel blocks the cells in the late G2 and M phases of the cell cycle (5), and, as shown in Fig. 2A-C, exposure to paclitaxel induced a G2/M block in all three cell lines. Notably, *SLC25A43* KD resulted in a significantly higher G2/M block at 16 nM paclitaxel in MCF7 and BT-474 compared to control. This, however, was not seen in MCF10A cells. The significant difference in G2/M distribution remained in MCF7 (P<0.05) and BT-474 (P<0.05) cells at the higher concentration of paclitaxel.

Epirubicin induces permanent double strand breaks leading to G2/M block (7,8). Similar to paclitaxel, epirubicin also induced G2/M block in the cell lines upon exposure (Fig. 2A-C). However, only MCF7 cells showed a significant change in cell cycle phase distribution between siCtrl and *SLC25A43* KD at the lower concentration (2.5 μM) of epirubicin. Exposure of the cell lines to 10 μM of epirubicin resulted in less G2/M block compared to 2.5 μM, but there was a significantly higher fraction of cells in G2/M phase in *SLC25A43* KD compared to control in both MCF7 (P<0.05) and BT-474 (P<0.05) cells.

Next, we examined the effects of trastuzumab on the cell cycle phase distribution after KD of *SLC25A43*. Trastuzumab is known to exhibit growth arrest in the G1 phase, partly due to inhibition of proteins involved in regulating the cell cycle (11–15). This was also confirmed in the BT-474 cells (Fig. 2D). Furthermore, KD of *SLC25A43* leads to a significantly decreased G1 block at both 10 and 100 μg/ml of trastuzumab (P<0.05). This result suggests that KD of *SLC25A43* causes a reduced sensitivity to the inhibitory mechanisms of trastuzumab, which correlates with the results regarding the reduced inhibition of proliferation in *SLC25A43* KD cells compared to control, following trastuzumab treatment (Fig. 1D). Exposure to trastuzumab in combination with paclitaxel resulted in reduced G2/M block compared to paclitaxel alone, however, the significant effect of *SLC25A43* KD on the G2/M block remained (Fig. 2C and D). These results demonstrate that KD of *SLC25A43* in combination with drug exposure alters the cell cycle phase distribution in the breast cancer cell lines.

### Ki-67 expression is altered by paclitaxel and epirubicin exposure

Loss of growth control, including aberrations in the mechanisms regulating the integrity of cell cycle progression, is a hallmark of cancer (27). Determining the expression of cell proliferation markers in tumour has therefore become increasingly important in the clinic (31,32). One marker of interest is Ki-67, a protein present in all phases of the cell cycle except in the G0 and the early G1 phase (33). In light of this and regarding the findings that KD of *SLC25A43* alters the cell cycle distribution upon drug exposure, we further investigated the effects of *SLC25A43* KD on the Ki-67 expression.

**Figure 3:**
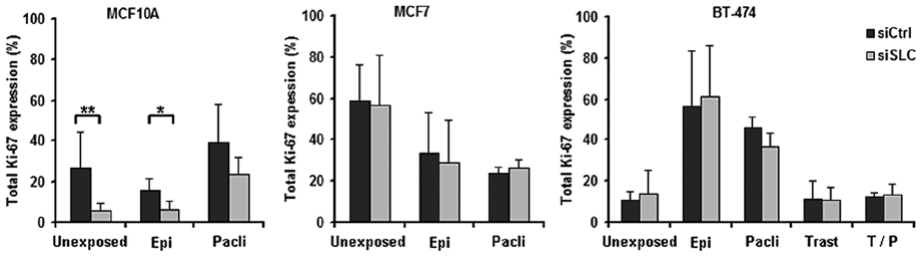
*SLC25A43* KD reduces the expression of proliferation marker Ki-67 in non-malignant MCF10A cells after epirubicin exposure. Total Ki-67 expression in MCF10A, MCF7 and BT-474 cells 72 h after exposure with 16 nM paclitaxel or 2.5 μM epirubicin, comparing siCtrl and siSLC-transfected cells. BT-474 cells were also exposed to 10 μg/ml trastuzumab or trastuzumab-paclitaxel double exposure (T/P) (16 nM and 10 μg/ml) for 72 h. The total expression of Ki-67 was only significantly altered in MCF10A cells after *SLC25A43* KD. For all assays, two experiments were performed in duplicates. The data are expressed as the means ± SD. ^*^P<0.05 and ^**^P<0.01.

**Figure 4:**
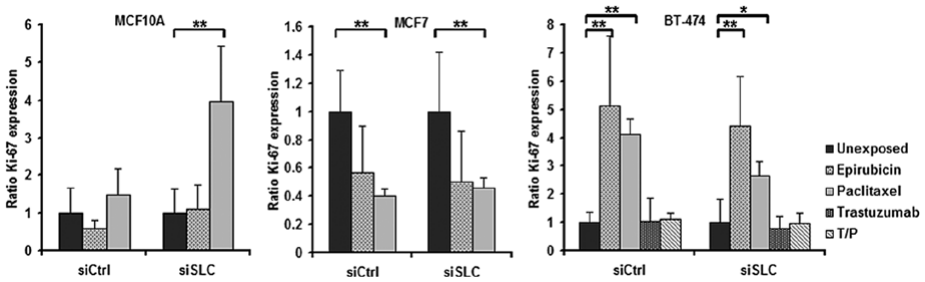
Ki-67 expression is altered after drug exposure. The graphs show the ratio of Ki-67 expression in MCF10A, MCF7 and BT-474 cells comparing unexposed and drug-exposed siCtrl or siSLC cells. The cells were exposed to 16 nM paclitaxel or 2.5 μM epirubicin for 72 h. BT-474 cells were also exposed to 10 μg/ml trastuzumab or trastuzumab-paclitaxel double exposure (T/P) (16 nM and 10 μg/ml) for 72 h. In MCF10A cells, the ratio of Ki-67 expression was only significantly increased after paclitaxel exposure in siSLC. In MCF7 cells, the ratio of Ki-67 expression was significantly lower in both siCtrl and siSLC after paclitaxel exposure. In BT-474 cells, both epirubicin and paclitaxel significantly increased the ratio of Ki-67 expression in both siCtrl and siSLC. For all assays, two experiments were performed in duplicates. The data are expressed as the means ± SD. ^*^P<0.05 and ^**^P<0.01.

In MCF10A cells, KD of *SLC25A43* led to a decreased total percentage of Ki-67-positive cells compared to siCtrl both in unexposed- and in epirubicin-exposed cells (Fig. 3). When exposing the cell lines to paclitaxel, the fraction of Ki-67-positive cells was found not to be altered by KD of *SLC25A43* when compared to siCtrl. However, paclitaxel was shown to influence the Ki-67 expression in all three cell lines (Fig. 4). In addition, exposure to epirubicin resulted in an increased fraction of Ki-67-positive BT-474 cells (Fig. 4).

As the mitochondrion has been demonstrated to impact the cell cycle regulation (20–26), we investigated if KD of *SLC25A43* in the three cell lines, when exposed to drugs, would contribute to changes in cell cycle regulation by analysis of p21 positivity. In our study, *SLC25A43* KD and drug exposure did not influence the p21 positivity in any of the three cell lines (data not shown).

## Discussion

The theory that mitochondria are important players of apoptosis, cell proliferation and cell cycle regulation (20–26) has expanded the field of cancer research, and it is now well established that mitochondrial dysfunction plays a key role in tumour development and response to therapy (27).

We previously demonstrated a common loss of the mitochondrial gene *SLC25A43* in HER2-positive breast cancer (28). We further showed that HER2-positive breast tumours with a lower expression of SLC25A43 also have a lower S phase fraction. These previous findings led to the hypothesis that an altered mitochondrial function through a reduced expression of SLC25A43 may alter the efficacy of antitumour drugs *in vitro*.

Using siRNA, we investigated the effects of *SLC25A43* knockdown (KD) on drug cytotoxicity in immortalised mammary epithelial cells, HER2-negative, and HER2-positive breast cancer cells after exposure to different drugs. In our study, KD of *SLC25A43* did not alter the viability following epirubicin exposure in any of the cell lines. This suggests that the mechanism of action of epirubicin cytotoxicity is, at least in part, independent of altered mitochondrial function due to *SLC25A43* KD. Paclitaxel exerts is cytotoxic effects through other mechanisms, involving microtubule assembly, cell cycle arrest and the mitochondrial apoptotic pathway (34–36). Deregulations in this pathway and other mitochondrial functions have been suggested to induce paclitaxel resistance (36–38). In our experiments, exposure of the HER2-negative MCF7 cells and the HER2-positive BT-474 cells to paclitaxel after KD increased the viability of the cells compared to control. This change in viability was accompanied by an increased G2/M block in the exposed KD cells, indicating that further blocking of the cells in G2/M may be beneficial for cell survival. Tan *et al*(39) demonstrated that delaying cell entry into M phase conferred survival advantages following paclitaxel exposure in breast cancer cells. BT-474 cells were further subjected to exposure to trastuzumab. KD of *SLC25A43* followed by exposure to trastuzumab resulted in decreased inhibitory effect on proliferation of the antibody and reduced the G0/G1 arrest. Trastuzumab is known to increase the association between p27^Kip1^ and CDK2 complexes resulting in induced G1 cell cycle arrest (11–15,40). Altered expression of these proteins has been connected with trastuzumab resistance (41). Our findings show that KD of *SLC25A43* reduces the G0/G1 arrest, possibly leading to the reduced inhibitory effect of trastuzumab in the HER2-positive BT-474 cells. When combining trastuzumab and paclitaxel to BT-474 cells, all survival and proliferative advantages due to *SLC25A43* KD previously observed are eradicated. This demonstrates the beneficial effect of dual exposure with cytostatic drugs and target therapies after *SLC25A43* KD.

Ki-67 is widely used as a proliferation marker in the clinic and it has also been shown to be a prognostic marker (31); however, studies investigating the predictive value of Ki-67 for chemotherapy present inconclusive data (42–45). When investigating the Ki-67 expression in the cell lines after drug exposure, *SLC25A43* KD was shown to influence the expression in the non-malignant MCF10A cells only after epirubicin exposure. However, both epirubicin and paclitaxel were found to alter the Ki-67 expression in the two breast cancer cell lines when compared to unexposed cells. MCF7 cells showed a decreased Ki-67 positivity while BT-474 cells showed an increased Ki-67 positivity following paclitaxel exposure. These findings may not be a direct result of the drug exposure; instead, it is more likely to be a secondary finding following the cell cycle arrest. It has previously been described in a clinical study that the Ki-67 expression was altered after chemotherapy, compared to before treatment, and a decreased expression was associated with reduced disease-free survival in rectal carcinoma (46). Also, p21 expression has been shown to be altered after treatment (46,47). In our study, however, we were not able to assess any differences in p21 expression due to drug exposure. The clinical importance of an altered Ki-67 and/or p21 expression after chemotherapy for patient outcome remains to be further elucidated.

Collectively, we have demonstrated that SLC25A43 alters the efficacy of paclitaxel through increased viability and increased G2/M arrest. A reduced expression of *SLC25A43* also diminishes the antiproliferative effect of the target therapy trastuzumab. Our findings support the role of altered mitochondrial function in cancer and drug resistance.
